# Asa Hill

**Published:** 1875-03

**Authors:** 


ASA HILL, D. D. S., who died of heart disease in Norwalk, Conn., November 25th, 1874, also received his degree of D. D. S. in 1847, from the Baltimore College of Dental Surgery. He commenced the practice of Dentistry in his native place, Norwalk, and continued there up to the time of his death, respected by all who knew him as a Christian gentleman, and leading practitioner. Hill's Stopping, a plastic material for temporary fillings, of which he was the inventor, has made his name well known to the profession.

